# Ferroptosis in multiple myeloma: molecular mechanisms and therapeutic opportunities

**DOI:** 10.3389/fonc.2026.1897942

**Published:** 2026-08-05

**Authors:** Wanfu Jiang, Li Xu, Changling Zhu, Guangqiang Bai, Sihao Peng, Na Ma

**Affiliations:** 1 School of Medicine, Anhui University of Science and Technology, Huainan, China; 2 Department of Hematology, Anhui No. 2 Provincial People’s Hospital (Affiliated Hospital of Anhui Second Medical College), Hefei, China; 3 Graduate School, Bengbu Medical University, Bengbu, China; 4 The First Hospital of Anhui University of Science and Technology, Huainan, China; 5 Department of Radiology, Anhui No. 2 Provincial People’s Hospital (Affiliated Hospital of Anhui Second Medical College), Hefei, China

**Keywords:** drug resistance, ferroptosis, GPX4, iron metabolism, lipid peroxidation, multiple myeloma, SLC7A11, therapeutic target

## Abstract

Multiple myeloma (MM) is a hematologic malignancy driven by clonal plasma-cell proliferation and remains largely incurable owing to disease relapse and acquired drug resistance. Ferroptosis, an iron-dependent form of regulated cell death characterized by excessive lipid peroxidation, has emerged as a potential therapeutic target in cancer and is increasingly implicated in MM biology. Unlike apoptosis and other classical cell death pathways, ferroptosis is closely associated with dysregulated iron metabolism, oxidative stress, and lipid peroxidation, all of which are key features of MM biology. This review first outlines the molecular mechanisms of ferroptosis and then summarizes current evidence linking ferroptosis to MM progression, therapeutic resistance, and emerging therapeutic strategies. In addition, current therapeutic strategies targeting ferroptosis are discussed, including inhibition of system Xc−, direct targeting of GPX4, modulation of iron metabolism, and their potential synergistic effects with existing anti-myeloma therapies. Finally, the major challenges associated with clinical translation are highlighted, together with future directions for biomarker development and ferroptosis-based therapeutic strategies. Collectively, ferroptosis represents a promising therapeutic concept for exploiting redox and metabolic vulnerabilities in MM. Nevertheless, current knowledge is derived predominantly from preclinical studies, and successful clinical translation will require improved tumor selectivity, rigorous safety evaluation, and biomarker-guided patient stratification.

## 1 Introduction

Multiple myeloma (MM) is a hematologic malignancy characterized by the clonal expansion of plasma cells within the bone marrow and is clinically associated with bone destruction, renal impairment, anemia, and immunodeficiency (1). Over the past two decades, advances in therapeutic strategies—such as proteasome inhibitors, immunomodulatory agents, and monoclonal antibodies—have significantly improved patient outcomes (1). Nevertheless, MM remains largely incurable because most patients ultimately relapse and develop resistance to current therapies (1). Globally, MM accounts for approximately 10% of hematologic malignancies and predominantly affects older adults, underscoring the continued need for novel therapeutic strategies (2).

MM pathogenesis is driven by both intrinsic genetic alterations and dynamic interactions with the bone marrow microenvironment (3). These interactions promote disease heterogeneity, metabolic adaptation, and therapeutic resistance, thereby limiting durable disease control (4, 5). Accumulating evidence indicates that dysregulated redox homeostasis and oxidative stress are fundamental features of MM biology and influence both disease progression and therapeutic response (6). These features provide a rationale for investigating regulated cell-death pathways that may exploit metabolic vulnerabilities in MM.

Ferroptosis is an iron-dependent form of regulated cell death driven by excessive lipid peroxidation and is mechanistically distinct from apoptosis, necroptosis, and autophagy (7). It is characterized by excessive accumulation of lipid reactive oxygen species together with failure of antioxidant defense systems, particularly those involving glutathione and glutathione peroxidase 4 (GPX4) (8). MM cells display substantial redox and metabolic heterogeneity, and available experimental evidence suggests that ferroptotic susceptibility is context dependent rather than uniform across disease subtypes or treatment states (9). Preclinical studies indicate that ferroptosis contributes to MM pathogenesis and may represent a therapeutically exploitable vulnerability (10).

This review provides a comprehensive overview of the molecular mechanisms governing ferroptosis, summarizes current evidence linking ferroptosis to MM progression and therapeutic resistance, and critically evaluates the translational potential of ferroptosis-targeted therapeutic strategies (11). Rather than merely summarizing canonical ferroptosis pathways and candidate ferroptosis-inducing agents, this review emphasizes MM-specific determinants of ferroptosis susceptibility. Particular attention is given to the relationship between ferroptosis and therapeutic sensitivity or resistance to proteasome inhibitors, immunomodulatory drugs, monoclonal antibodies, and emerging cellular immunotherapies. By distinguishing mechanistic insights from preclinical therapeutic findings and currently available clinical evidence, the present review provides a balanced assessment of the translational potential, current limitations, and future directions of ferroptosis-targeted strategies in MM. This review further highlights MM-specific ferroptosis regulation by the bone marrow microenvironment, myeloma-associated bone disease, treatment-induced adaptation, and therapeutic resistance.

## 2 Pathogenesis of multiple myeloma

### 2.1 Genetic alterations in MM

Genetic abnormalities contribute to MM initiation, clonal evolution, and disease progression. The hallmark events are IgH translocations, such as t (11;14) upregulating CCND1, t(4;14) affecting FGFR3/NSD2, and t(14;16) involving MAF (12). These translocations juxtapose oncogenes with immunoglobulin enhancer elements, thereby promoting plasma-cell transformation (13). Besides translocations, copy number alterations are common—gain(1q) and del(17p) correlate with rapid progression and short survival (14). Single-cell DNA and RNA sequencing studies have demonstrated substantial clonal heterogeneity across disease stages (15). KRAS and NRAS mutations are detected in nearly half of patients, frequently coexisting with NF-κB activating lesions (16). TP53 mutations, though rare at diagnosis, emerge at relapse and drive therapy resistance (17). Beyond coding mutations, epigenetic modifiers like KDM6A, HIST1H1E, and CREBBP are increasingly recognized; their disruption alters chromatin accessibility and contributes to aberrant gene expression without changing DNA sequence (18). Longitudinal studies indicate that treatment may select for subclones harboring specific mutations, thereby contributing to clonal selection. Importantly, recent work identified mutations in the RNA splicing machinery (e.g., SF3B1, U2AF2) in a subset of relapsed patients, suggesting alternative mechanisms of genomic instability (19). These genetic lesions not only drive proliferation but also shape drug responses—for instance, t (4;14) confers relative bortezomib sensitivity but poor outcomes with immunomodulatory drugs alone. These genetic alterations are clinically important for risk stratification and therapeutic decision-making. Their direct effects on ferroptosis susceptibility in MM remain largely unresolved; nevertheless, they may influence redox homeostasis, lipid metabolism, and treatment-induced stress responses, thereby providing a rationale for subtype-aware ferroptosis studies.

### 2.2 Dysregulated signaling pathways in MM

Beyond genetic alterations, aberrant intracellular signaling is critical for MM cell survival and drug resistance. The NF-κB pathway—both canonical (IKK complex) and non-canonical (NIK-dependent)—is constitutively active in most MM patients (20). It upregulates anti-apoptotic proteins like BCL-XL and MCL1, reducing sensitivity to chemotherapy. The PI3K/AKT pathway regulates metabolic adaptation and survival; it becomes rapidly activated upon adhesion to bone marrow stromal cells, contributing to cell adhesion-mediated drug resistance. MAPK signaling, often triggered by RAS or BRAF mutations, supports proliferation under hypoxic or nutrient-limited conditions. These pathways form an interconnected signaling network rather than functioning as isolated linear cascades. For example, KRAS mutations can simultaneously activate MAPK and PI3K/AKT; cross-talk between NF-κB and PI3K/AKT amplifies survival signaling. Pharmacological inhibition of one signaling pathway may induce compensatory activation of alternative survival pathways—MEK inhibitors can increase pAKT, limiting efficacy. Accordingly, therapeutic strategies targeting multiple signaling nodes or convergent downstream effectors are being evaluated clinically. Non-coding RNAs, including miR-21, miR-221, and lncRNA MALAT1, fine-tune these cascades. More recently, the Hippo pathway (YAP/TAZ) has been implicated in MM adhesion and survival. Several survival pathways active in MM, including NF-κB and PI3K/AKT signaling, may converge on antioxidant and metabolic programs that influence ferroptosis susceptibility. However, direct MM-specific evidence defining NF-κB-dependent regulation of GPX4 or SLC7A11 remains limited (21). A more comprehensive understanding of this signaling network may facilitate the development of strategies to overcome acquired therapeutic resistance.

### 2.3 Bone marrow microenvironment and MM progression

The bone marrow (BM) microenvironment provides a dynamic niche that can support MM-cell survival, disease progression, and reduced sensitivity to therapy. It includes stromal cells, osteoclasts, endothelial cells, and immune cells, all engaging in bidirectional crosstalk with malignant plasma cells (4). Stromal cells secrete IL-6, IGF-1, and BAFF, activating JAK/STAT3 and PI3K/AKT pathways to block apoptosis. Osteoclast-associated signaling, including TNF-α and RANKL, may further support MM cell survival and disease progression. Cell adhesion-mediated drug resistance (CAM-DR) is an important mechanism through which interactions with extracellular matrix components and stromal cells can reduce drug responsiveness in MM (22). Recent studies highlight metabolic reprogramming within the niche—MM cells induce lipolysis in adjacent adipocytes, acquiring free fatty acids for oxidative phosphorylation. Hypoxic regions stabilize HIF-1α, which rewires glucose metabolism and promotes angiogenesis via VEGF (23). Within the immune microenvironment, the accumulation of regulatory T cells and myeloid-derived suppressor cells, together with functional impairment of NK cells and CD8+ T cells, may contribute to immune evasion (24). Experimental findings also points to the gut–bone marrow axis: microbial metabolites (e.g., short-chain fatty acids) influence MM progression in preclinical models (25). Therapeutic approaches targeting the bone marrow niche, including cytokine blockade, chemokine receptor inhibition, and T-cell-engaging bispecific antibodies, are under investigation (26). Additionally, targeting extracellular matrix components (e.g., hyaluronan) or metabolic dependencies (e.g., inhibiting FAO) is under investigation (27). These microenvironment-directed approaches may complement direct anti-myeloma therapies. Microenvironmental cues such as hypoxia and stromal contact are also likely to impact ferroptosis sensitivity, as they fundamentally alter iron handling and redox balance in MM cells (28).

### 2.4 Bone marrow microenvironmental regulation of ferroptosis in MM

**Figure 1:**
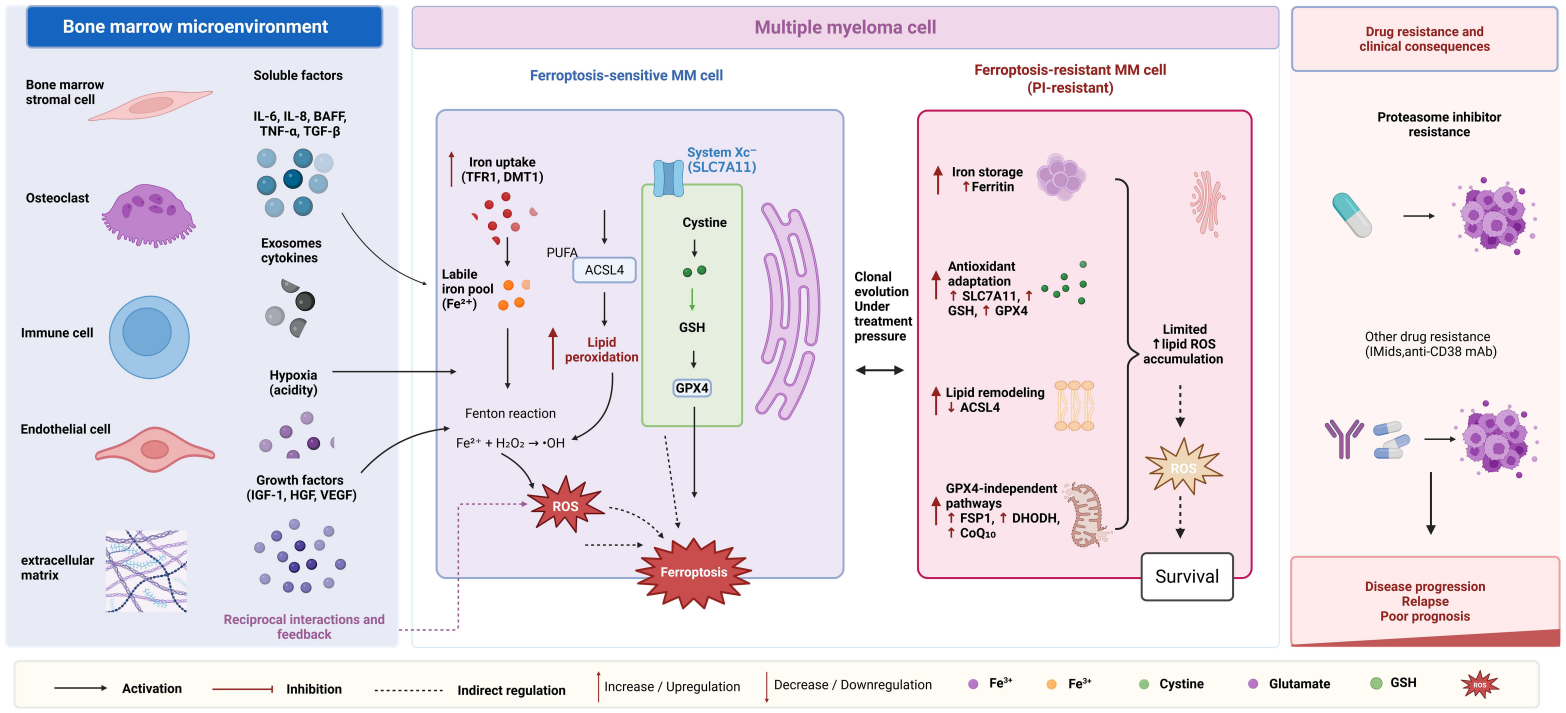
Bone marrow microenvironment and ferroptosis sensitivity in multiple myeloma. The bone marrow microenvironment provides soluble factors, growth factors, hypoxic and acidic conditions, and cellular interactions that may influence ferroptosis sensitivity in MM cells. In ferroptosis-sensitive MM cells, increased iron uptake and expansion of the labile iron pool promote Fenton chemistry, ROS generation, lipid peroxidation, and ferroptotic cell death. ACSL4-mediated incorporation of polyunsaturated fatty acids into membrane phospholipids, together with cystine uptake through system Xc^-^ (SLC7A11) and GPX4-dependent antioxidant defense, contributes to the ferroptosis threshold. Under therapeutic pressure, MM cells may acquire ferroptosis-resistant features, including increased iron storage, antioxidant adaptation, lipid remodeling, and activation of GPX4-independent protective pathways, thereby limiting lipid ROS accumulation and promoting survival. These mechanisms may contribute to proteasome inhibitor resistance in preclinical settings; however, their relevance to resistance to IMiDs, anti-CD38 antibodies, and other therapeutic modalities remains incompletely defined. Reciprocal interactions between MM cells and the bone marrow microenvironment may further shape ferroptosis susceptibility and therapeutic response.

The bone marrow microenvironment is a key determinant of MM progression and therapeutic resistance and may additionally modulate ferroptosis susceptibility. Stromal cells promote MM cell survival through direct adhesion and the secretion of cytokines such as interleukin-6, insulin-like growth factor-1, and CXCL12. These signals activate pro-survival pathways, including JAK/STAT3, PI3K/AKT, and NF-κB, which may enhance antioxidant capacity and resistance to oxidative stress. Although direct evidence remains limited, stromal-cell-mediated redox adaptation may increase the threshold for ferroptosis in MM cells (4). Hypoxia is a characteristic feature of the bone marrow niche. Hypoxia-inducible signaling can regulate mitochondrial activity, reactive oxygen species production, lipid metabolism, and glutathione-dependent antioxidant defenses (29). Its effect on ferroptosis is likely context dependent and remains insufficiently defined in MM. Myeloma-associated bone disease may also be relevant, as osteoclast activation, impaired osteoblast differentiation, and bone destruction are closely associated with oxidative stress and inflammatory signaling. Ferroptotic stress has been reported to impair osteoblast viability in other bone-related disease models, suggesting that ferroptosis induction may have both anti-myeloma potential and possible bone toxicity (30). The immune microenvironment may further modify ferroptosis-related responses. Ferroptotic tumor cells can release oxidized lipid mediators and damage-associated signals that influence immune activation, whereas excessive lipid peroxidation may impair T-cell and natural killer-cell function (31). This bidirectional influence should be considered when designing combinations of ferroptosis-targeted agents and immunotherapies. Furthermore, iron availability within the marrow niche may affect the labile iron pool of MM cells. Because renal impairment is common in MM and ferroptosis has been implicated in tubular injury, renal safety should be considered when developing systemic ferroptosis-inducing strategies (32) (**Figure 1**).

## 3 General features of ferroptosis

### 3.1 Iron metabolism and ferroptosis

Iron metabolism is a central determinant of ferroptosis initiation. Under physiological conditions, cellular iron homeostasis is maintained through coordinated regulation of iron uptake, storage, utilization, and export. Disruption of this balance can increase the intracellular labile iron pool and promote oxidative damage. Labile ferrous iron (Fe²^+^) catalyzes the Fenton reaction, generating highly reactive hydroxyl radicals from hydrogen peroxide and thereby promoting membrane lipid peroxidation (33, 34). Key players in iron handling include transferrin and its receptor TFR1 (iron uptake), ferritin (storage), and ferroportin (export). Alterations in these processes can expand the labile iron pool and increase cellular susceptibility to ferroptosis. For example, knocking down ferritin heavy chain increases free iron and sensitizes cancer cells to ferroptosis inducers (35). Recent work has highlighted a process called ferritinophagy—selective autophagic degradation of ferritin mediated by NCOA4. When NCOA4 is upregulated, ferritin breaks down, releasing iron and boosting lipid peroxidation (36, 37). This mechanism provides a direct molecular link between autophagy and ferroptosis. In MM, iron metabolism represents a potentially relevant therapeutic vulnerability, although the clinical significance of individual iron-handling proteins remains incompletely defined (38). Moreover, iron chelators like deferoxamine block ferroptosis, while iron supplementation accelerates it. These iron-regulatory processes may provide a rationale for evaluating combinations of iron-loading approaches and GPX4 inhibition in preclinical cancer models.

### 3.2 Lipid peroxidation in ferroptosis

Lipid peroxidation constitutes the terminal effector mechanism of ferroptosis, disrupting membrane integrity. Lipid peroxidation is initiated by oxidation of polyunsaturated fatty acid (PUFA)-containing phospholipids, leading to the formation of lipid hydroperoxides that subsequently decompose into reactive aldehydes such as 4-HNE and MDA (39). ACSL4 serves as a key determinant of ferroptosis susceptibility; it activates free PUFAs (especially arachidonic acid and adrenic acid) and helps incorporate them into membrane phospholipids (40). Loss of ACSL4 markedly reduces cellular susceptibility to ferroptosis. Following their incorporation into membrane phospholipids, lipoxygenases (LOXs) directly add oxygen to them, initiating chain reactions. Importantly, non-enzymatic free radical propagation also contributes substantially to lipid peroxidation, allowing oxidative damage to spread in a self-amplifying manner unless terminated by antioxidant defense systems. Available data points to other enzymes that can promote lipid peroxidation, including cytochrome P450 oxidoreductase (POR) and certain NADPH oxidases (NOXs). In cancer cells, the balance between PUFA synthesis (via fatty acid desaturases like FADS2) and degradation determines ferroptosis sensitivity. Also, Membrane composition also influences ferroptosis susceptibility—mitochondrial membranes, with their high content of cardiolipin (a specialized PUFA-rich lipid), can be primary targets. In multiple myeloma, ACSL4 expression varies across patients; higher levels may predict response to ferroptosis-inducing therapies. Measuring lipid peroxidation directly, for instance using the C11-BODIPY probe, is now routine in ferroptosis research (41).

### 3.3 The SLC7A11–GSH–GPX4 antioxidant axis

This is the canonical and most extensively characterized defense system against ferroptosis. At its core is GPX4, a selenoprotein that directly reduces lipid hydroperoxides to harmless lipid alcohols (42). In the absence of GPX4, lipid peroxides accumulate rapidly and trigger ferroptotic cell death. GPX4 depends on glutathione (GSH) as its electron donor. GSH is a tripeptide (cysteine–glutamate–glycine) synthesized in two steps, with cysteine availability being the limiting factor. Cells take up cystine (oxidized cysteine dimer) through the system Xc^-^ antiporter, which comprises SLC7A11 (the light chain) and SLC3A2 (the heavy chain). SLC7A11 exchanges extracellular cystine for intracellular glutamate (43). Once inside, cystine is reduced to cysteine and used for GSH synthesis. Many cancer cells, including multiple myeloma, upregulate SLC7A11 to buffer oxidative stress. Inhibiting system Xc^-^ with erastin or sulfasalazine depletes GSH, inactivates GPX4, and triggers ferroptosis (44). However, direct GPX4 inhibitors like RSL3 are even more potent. Recent work has revealed that GPX4 degradation via the ubiquitin-proteasome pathway (e.g., by TRIM25 or HERC5) can also sensitize cells (45). Interestingly, some myeloma cells with high baseline GPX4 expression are intrinsically resistant to ferroptosis. Combining GPX4 inhibition with iron loading or with drugs that deplete cysteine (e.g., cyst(e)inase) is being explored in preclinical models (46).

### 3.4 Emerging GPX4-independent ferroptosis defense pathways

**Figure 2:**
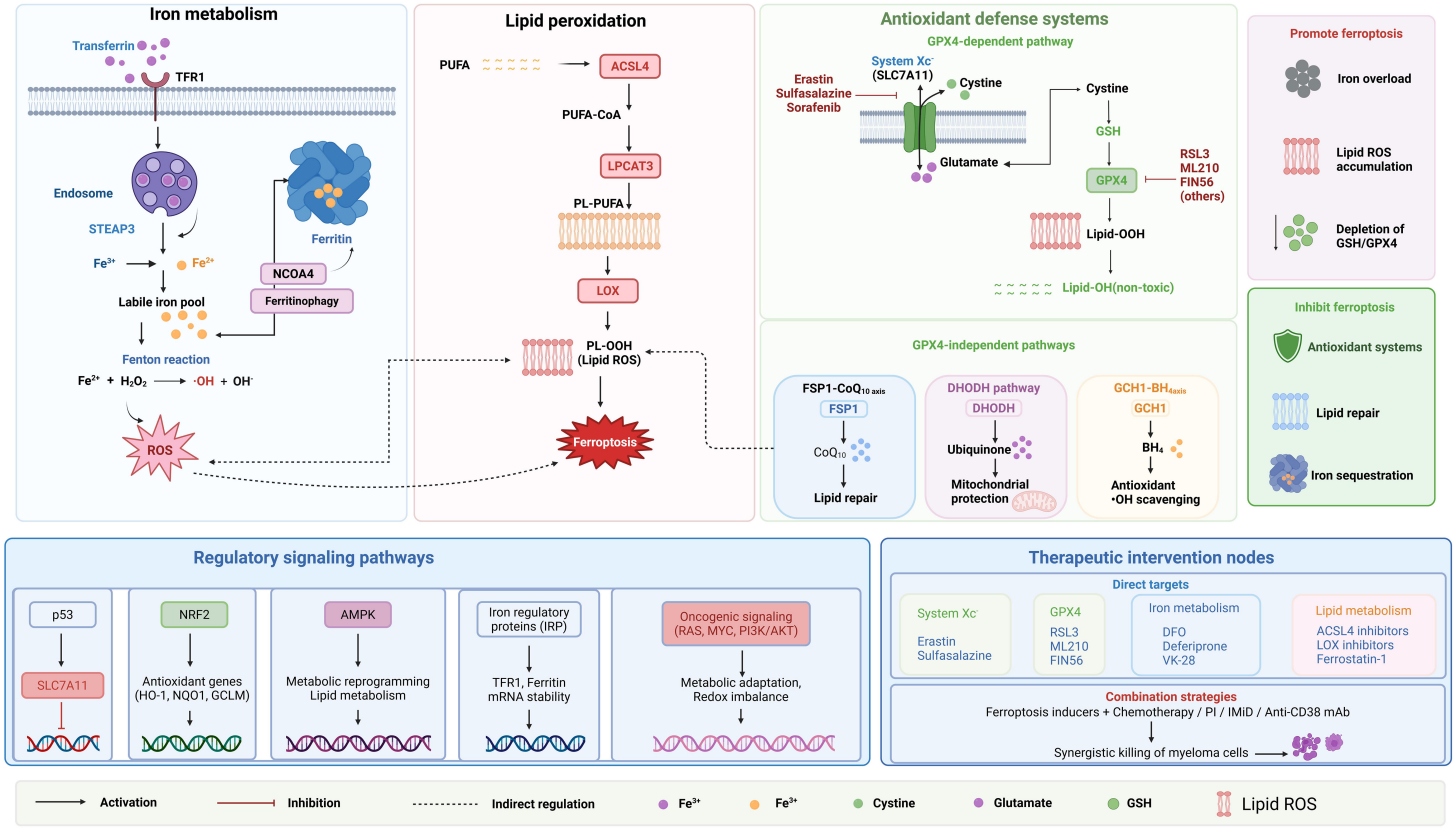
Molecular mechanisms, regulatory pathways, and therapeutic intervention nodes of ferroptosis in multiple myeloma (MM). Schematic overview of ferroptosis regulation in MM, including iron metabolism, lipid peroxidation, GPX4-dependent antioxidant defense, and GPX4-independent pathways involving FSP1, DHODH, and GCH1. Iron overload and ferritinophagy increase intracellular labile iron and reactive oxygen species (ROS), thereby promoting lipid peroxidation and ferroptosis. The classical SLC7A11–GSH–GPX4 axis suppresses ferroptosis by detoxifying lipid hydroperoxides, whereas GPX4-independent antioxidant systems provide parallel protective mechanisms. Regulatory signaling pathways, including NRF2, AMPK, p53, oncogenic signaling, and iron regulatory proteins, further modulate ferroptosis sensitivity. Potential ferroptosis-related intervention nodes, including system Xc^-^ inhibition, GPX4 targeting, iron metabolism modulation, and combination approaches with anti-myeloma therapies, are illustrated. Most of these intervention strategies remain preclinical or mechanistic concepts in MM and require further validation.

Not all ferroptosis resistance depends on GPX4. Over the past few years, several parallel systems have been identified, each providing additional protective mechanisms. The best characterized is FSP1 (ferroptosis suppressor protein 1, also known as AIFM2). FSP1 is a NAD(P)H-dependent oxidoreductase that reduces coenzyme Q10 (CoQ10) to ubiquinol (CoQ10H_2_), a lipophilic radical-trapping antioxidant that works in membranes independently of GPX4 (47). FSP1 can fully compensate for GPX4 loss in some cell lines. Another mitochondrial pathway involves DHODH (dihydroorotate dehydrogenase), which produces CoQ10H_2_ specifically in the inner mitochondrial membrane, protecting mitochondria from ferroptotic damage (48). The third pathway is GCH1 (GTP cyclohydrolase 1), which generates tetrahydrobiopterin (BH4), another potent radical scavenger that guards phospholipids (49). More recently, the NRF2–p62–Keap1 axis has been shown to regulate several of these defense genes, linking metabolic stress to ferroptosis resilience (50). The MM-specific contribution of GPX4-independent defense pathways, including FSP1, DHODH, and GCH1, remains insufficiently defined and should be considered a priority for future investigation. These GPX4-independent mechanisms not only expand our understanding but also offer new drug targets. Because cancer cells can switch between defense systems, targeting two parallel pathways simultaneously may be required to overcome therapy resistance. Targeting these parallel defense systems may therefore represent an effective strategy to overcome ferroptosis resistance in MM (**Figure 2**; **Table 1**).

**Table 1:** Ferroptosis-related pathways in multiple myeloma: MM-specific evidence and remaining uncertainties.

Pathway	Key molecules	Evidence in MM	Potential relevance	Evidence level	Key references
Iron mobilization and ferritinophagy	NCOA4, ferritin, Fe²^+^	Bortezomib increased intracellular free Fe²^+^ through NCOA4-mediated ferritinophagy and enhanced RSL3 activity in MM cells.	Iron mobilization may sensitize MM cells to ferroptotic stress.	MM cell-based and preclinical evidence	(38)
System Xc^-^–GSH–GPX4 axis	SLC7A11, GSH, GPX4	SLC7A11–GSH–GPX4 signaling is mechanistically relevant to ferroptosis; high SLC7A11 expression may indicate dependence on cystine uptake in selected MM models.	Potential vulnerability to system Xc^-^ or GPX4 inhibition; prognostic utility remains unvalidated.	Experimental and exploratory transcriptomic evidence	(53, 54)
Lipid peroxidation susceptibility	ACSL4, PUFA-phospholipids	ACSL4 is a canonical determinant of ferroptosis sensitivity, but its MM-specific expression pattern and functional role remain heterogeneous.	Requires subtype-specific validation before therapeutic targeting.	General ferroptosis mechanism; limited MM-specific evidence	(54, 68)
GPX4-independent defense pathways	FSP1, DHODH, GCH1/BH4	These pathways suppress ferroptosis in other cancer models; direct functional validation in MM remains limited.	Candidate mechanisms for future MM studies, not validated MM therapeutic targets.	General mechanistic evidence	(19, 49, 65)
NRF2/HO-1-associated redox regulation	NRF2, KEAP1, HMOX1	NRF2/HO-1-related ferroptosis modulation has been reported in MM pharmacological studies.	May influence redox adaptation and sensitivity to ferroptosis-inducing compounds.	MM cell-based preclinical evidence	(85–87)
Mitochondrial stress–innate immune signaling	TFAM, cGAS–STING, SLC7A11	Zalcitabine induced ferroptosis through a TFAM–cGAS–STING–SLC7A11 axis in MM models.	Supports mitochondrial stress signaling as a candidate combination strategy.	MM cell-based and xenograft evidence	(84)
STK17B-associated ferroptosis regulation	STK17B, labile iron pool, lipid ROS	STK17B inhibition increased labile iron and lipid peroxidation and sensitized MM models to therapy.	Candidate approach for resistance-associated MM; requires further validation.	MM cell-based and xenograft evidence	(10)

## 4 Biological relevance and therapeutic vulnerability of ferroptosis in MM

### 4.1 Dysregulated iron metabolism in MM

Iron homeostasis is frequently disrupted in MM, resulting in an expanded labile iron pool that primes cells for ferroptosis (51). MM cells exhibit altered iron uptake and intracellular iron handling, which influence their susceptibility to ferroptotic stress. TFR1 (transferrin receptor 1) is frequently upregulated, whereas ferroportin is downregulated, thereby promoting intracellular iron accumulation (52). Excess ferrous iron subsequently drives Fenton reactions, generating hydroxyl radicals that initiate membrane lipid peroxidation. Although moderate levels of reactive oxygen species (ROS) support MM-cell proliferation, excessive iron-dependent oxidative stress can trigger ferroptosis. Recent studies have identified additional regulatory mechanisms governing iron metabolism in MM. Heme oxygenase-1 (HO-1) releases ferrous iron through heme degradation, thereby providing an additional source of intracellular labile iron (9). Collectively, these regulatory mechanisms suggest that modulation of iron homeostasis may shift oxidative stress from a tumor-supportive state toward ferroptosis-mediated cell death. Published studies demonstrate that bortezomib increases intracellular free Fe²^+^ through NCOA4-mediated ferritinophagy, thereby enhancing the anti-myeloma activity of RSL3 and supporting iron mobilization as a preclinical ferroptosis-sensitizing strategy in MM (38).

### 4.2 Ferroptosis-related gene expression and prognostic implications in MM

Over the past few years, increasing attention has been directed toward the expression profiles and prognostic relevance of ferroptosis-related genes in MM. Key regulators include GPX4, the major phospholipid hydroperoxide detoxifying enzyme; SLC7A11, the cystine transporter subunit that supports glutathione synthesis; and ACSL4, which promotes the incorporation of polyunsaturated fatty acids into membrane phospholipids. Altered expression of ferroptosis-related genes has been explored in MM transcriptomic datasets and experimental models; however, the expression patterns and clinical implications of individual genes remain insufficiently validated (53). In particular, SLC7A11 may represent a context-dependent vulnerability rather than a uniformly protective factor. A recent MM study showed that cells with high SLC7A11 expression were sensitive to erastin-induced ferroptosis, indicating that elevated cystine transport may coexist with increased dependence on the SLC7A11–glutathione antioxidant system (54). Accordingly, SLC7A11 expression alone should not be interpreted as a validated marker of ferroptosis resistance or poor prognosis in MM.ACSL4 is a canonical determinant of ferroptosis sensitivity in several tumor types because it facilitates the generation of peroxidation-prone phospholipids. Nevertheless, its expression and functional significance appear heterogeneous across MM datasets and have not yet been prospectively linked to clinical outcomes. Similarly, although GPX4 is widely recognized as a central ferroptosis-defense enzyme, direct evidence establishing GPX4 expression as an independent prognostic biomarker or treatment-response predictor in MM remains limited. Recent bioinformatic studies have identified ferroptosis-related gene signatures that stratify MM patients into distinct risk groups and may be associated with immune-cell infiltration, survival outcomes, and predicted drug sensitivity (53, 55). However, these signatures were largely developed from retrospective public datasets and require validation in independent prospective cohorts, ideally together with functional confirmation in patient-derived MM cells. Thus, ferroptosis-related genes should currently be regarded as exploratory biomarkers rather than clinically actionable predictors in MM (53, 55) (**Table 2**).

**Table 2:** Candidate ferroptosis-related biomarkers and research applications in multiple myeloma.

Candidate marker or signature	Current MM evidence	Possible research application	Main limitation	Key references
SLC7A11	High SLC7A11 expression was associated with sensitivity to erastin-induced ferroptosis in an MM study.	May identify dependence on cystine uptake and antioxidant buffering.	Expression alone does not establish prognosis or ferroptosis resistance.	(54)
GPX4	Central ferroptosis-defense enzyme; its independent prognostic value in MM has not been established.	Pharmacodynamic marker of lipid-peroxide detoxification.	Limited prospective MM validation.	(53)
ACSL4	Canonical regulator of PUFA-phospholipid metabolism; MM-specific clinical relevance remains uncertain.	Candidate indicator of ferroptosis-prone lipid states.	Heterogeneous expression and limited functional validation in MM.	(40, 53)
NCOA4/ferritinophagy activity	Linked to bortezomib-associated iron mobilization and RSL3 sensitization in MM cells.	Potential pharmacodynamic marker for iron-mobilizing combination strategies.	Not validated in patient samples.	(38)
NRF2/HO-1-related signaling	Pharmacological modulation of NRF2/HO-1 has induced ferroptosis in MM models.	Candidate marker of redox adaptation.	Compound-specific findings; no clinical cut-off or predictive validation.	(85–87)
Ferroptosis-related gene signatures	Retrospective transcriptomic signatures have stratified MM cohorts into risk groups.	Hypothesis generation for risk stratification and therapeutic response studies.	Retrospective datasets; requires external prospective and functional validation.	(53)

### 4.3 Ferroptosis as a therapeutic vulnerability in MM

MM cells exhibit constitutively elevated ROS levels that promote proliferation; however, they rely on robust antioxidant defenses to prevent excessive oxidative damage. This intrinsic oxidative state creates a therapeutic vulnerability, whereby further enhancement of lipid peroxidation or disruption of antioxidant defenses can selectively eliminate MM cells. Several studies have demonstrated that ferroptosis inducers are effective against MM cells, including those that are resistant to proteasome inhibitors (PIs) (38). The mechanistic association between PI resistance and ferroptosis is primarily attributed to adaptive alterations in redox homeostasis and lipid metabolism. Bortezomib-resistant cells frequently exhibit increased NRF2 activity, leading to upregulation of GPX4 and SLC7A11, while simultaneously accumulating higher levels of labile iron and polyunsaturated fatty acids (PUFAs) (56). Collectively, adaptive mechanisms that confer resistance to proteasome inhibition simultaneously increase cellular dependence on ferroptosis-defense pathways, thereby creating a therapeutic vulnerability to ferroptosis induction. Preclinical studies have demonstrated that erastin (an SLC7A11 inhibitor) and RSL3 (a GPX4 inhibitor) significantly reduce the viability of MM cell lines and primary patient-derived samples. Combination strategies have demonstrated encouraging therapeutic efficacy in preclinical MM models. For example, erastin combined with bortezomib exhibits synergistic antitumor activity both *in vitro* and in xenograft models. In addition, combining GPX4 inhibitors with iron-loading agents, such as ferric ammonium citrate, further enhances ferroptosis induction and antitumor activity. More recently, liposomal formulations of RSL3 have been developed to improve drug delivery and reduce off-target toxicity (57). Another emerging area of investigation involves the immune microenvironment. Ferroptotic MM cells release damage-associated molecular patterns (DAMPs), which may promote dendritic-cell activation and T-cell-mediated immune responses; however, whether ferroptosis enhances antitumor immunity in MM remains to be established (58). Several early-phase clinical studies are evaluating ferroptosis-related therapeutic strategies, including sulfasalazine, in hematologic malignancies; however, clinical evidence remains preliminary (59). Future studies should prioritize the rational integration of ferroptosis inducers with established anti-myeloma therapies while incorporating biomarkers of iron metabolism and lipid peroxidation to optimize patient selection and therapeutic efficacy.

## 5 Ferroptosis and drug resistance in MM

### 5.1 Antioxidant adaptation and ferroptosis resistance

Drug-resistant MM cells not only evade the cytotoxic effects of therapy but also undergo extensive remodeling of their antioxidant defense networks. These cells maintain elevated levels of reactive oxygen species (ROS) to support proliferation, whereas resistant clones markedly upregulate antioxidant systems to limit oxidative damage. The most common adaptive mechanism involves upregulation of the SLC7A11–glutathione–GPX4 axis. Upregulation of SLC7A11 enhances cystine uptake, glutathione synthesis, and consequently GPX4 activity, thereby facilitating the detoxification of lipid peroxides (60). This antioxidant buffering capacity maintains lipid ROS below cytotoxic thresholds, even under therapeutic stress. Experimental findings indicate that resistant MM cells also upregulate FSP1 and GCH1 as compensatory antioxidant systems, suggesting that pharmacological inhibition of GPX4 alone may be insufficient to induce ferroptosis (61). Hyperactivation of the transcription factor NRF2, resulting from KEAP1 mutations or epigenetic alterations, represents an important mechanism underlying antioxidant adaptation in resistant MM cells (62). Some MM cells acquire ferroptosis resistance without increasing GPX4 expression. Instead, they remodel membrane lipid composition by replacing polyunsaturated fatty acids (PUFAs) with less oxidation-prone monounsaturated fatty acids (MUFAs) through upregulation of stearoyl-CoA desaturase 1 (SCD1) (63). This lipid remodeling decreases susceptibility to lipid peroxidation and thereby enhances resistance to ferroptosis. Autophagy also contributes to ferroptosis regulation. Ferritinophagy promotes ferroptosis by releasing intracellular iron, whereas resistant MM cells may suppress ferritinophagy to limit iron availability (64). Collectively, antioxidant adaptation in MM is mediated by multiple complementary mechanisms rather than a single signaling pathway. Identifying the dominant adaptive mechanism in individual resistant clones may facilitate the rational selection of combination therapeutic strategies. In addition to GPX4-dependent antioxidant defenses, GPX4-independent pathways, including the FSP1-mediated system, may also contribute to ferroptosis resistance in MM (65).

### 5.2 Ferroptosis vulnerabilities in proteasome inhibitor-resistant MM

Resistance to bortezomib and other proteasome inhibitors (PIs) remains a major clinical challenge in MM. Emerging evidence indicates that ferroptosis-related pathways are mechanistically associated with proteasome inhibitor resistance in MM. PI-resistant cells exhibit enhanced redox buffering capacity and increased metabolic flexibility. These cells frequently exhibit altered lipid metabolism, including a shift away from polyunsaturated fatty acid (PUFA)-rich membrane phospholipids, thereby reducing basal lipid peroxidation (66). These metabolic adaptations are generally associated with reduced susceptibility to ferroptosis, although this effect is context dependent. However, these adaptive redox and metabolic alterations also create distinct therapeutic vulnerabilities. PI-resistant cells frequently accumulate higher levels of labile iron and exhibit increased mitochondrial ROS production (38). They also become highly dependent on specific ferroptosis-defense pathways, particularly GPX4- and FSP1-mediated antioxidant systems, for survival (65). Preclinical investigations suggest that treatment of PI-resistant MM cells with GPX4 inhibitors (e.g., RSL3 and ML162) or the FSP1 inhibitor iFSP1 induces extensive lipid peroxidation and ferroptotic cell death, even in cells that are highly resistant to bortezomib (65). These findings are consistent with a synthetic lethal interaction, whereby the metabolic rewiring that confers PI resistance simultaneously renders ferroptosis-defense pathways essential for cell survival. Moreover, single-cell analyses have shown that, within PI-resistant populations, cells with higher GPX4 expression are particularly sensitive to GPX4 inhibition (66). Another important observation is that combining low-dose bortezomib, which further increases endoplasmic reticulum stress, with a sublethal dose of a ferroptosis inducer may selectively enhance ferroptotic cell death in resistant MM cells without substantially increasing toxicity to normal cells (38). Clinical samples obtained from relapsed MM patients following bortezomib treatment exhibit increased GPX4 and SLC7A11 expression, suggesting that ferroptosis-defense pathways may undergo positive selection during therapeutic resistance (39). Targeting these pathways may convert therapeutic resistance into increased susceptibility to ferroptosis. Collectively, these findings identify ferroptosis as a therapeutically actionable vulnerability for overcoming proteasome inhibitor resistance in MM.

### 5.3 Ferroptosis induction as a strategy to overcome resistance

Based on these vulnerabilities, pharmacological induction of ferroptosis represents an attractive strategy for eliminating drug-resistant MM cells. Preclinical studies have demonstrated that ferroptosis inducers, including erastin (a system Xc^-^ inhibitor) and RSL3 (a direct GPX4 inhibitor), effectively reduce the viability of PI-resistant MM cell lines and primary patient-derived samples (67). However, the greatest therapeutic potential is likely to reside in combination treatment strategies. Combining ferroptosis inducers with bortezomib or carfilzomib has demonstrated synergistic antitumor activity in multiple preclinical models (38). For example, erastin depletes intracellular glutathione (GSH), whereas bortezomib further increases ROS accumulation; together, these effects overwhelm the cellular antioxidant defense capacity (67). More recent studies have evaluated the dual targeting of GPX4 and FSP1 in MM, demonstrating that resistant cells frequently upregulate FSP1 following GPX4 inhibition. Co-administration of RSL3 and iFSP1, an FSP1 inhibitor, abolishes this compensatory mechanism and induces ferroptotic cell death in MM clones that survive single-agent treatment (68). Nanomedicine-based approaches have also been investigated. Encapsulation of RSL3 within liposomes or nanoparticles improves drug delivery to the bone marrow microenvironment while reducing systemic toxicity (69). Several studies have further evaluated FDA-approved drugs that exhibit ferroptosis-inducing off-target effects, including sulfasalazine, an SLC7A11 inhibitor, and statins, which reduce coenzyme Q10 (CoQ10) levels and may impair FSP1 function (70). Preliminary evidence suggests that these agents may be repurposed as adjunctive therapeutic strategies for MM. Combining ferroptosis inducers with conventional therapies may also delay the emergence of therapeutic resistance by eliminating resistant subclones that depend on specific antioxidant defense pathways (9). Several phase I/II clinical trials in hematologic malignancies are evaluating the combination of sulfasalazine and bortezomib; however, MM-specific clinical evidence remains limited (71). Collectively, these findings support the clinical potential of ferroptosis induction as a strategy for overcoming therapeutic resistance in MM. Thus, ferroptosis induction not only represents an alternative cytotoxic strategy but also provides a rational therapeutic approach for targeting key biological vulnerabilities in drug-resistant MM.

## 6 Therapeutic targeting of ferroptosis in MM

### 6.1 Ferroptosis-inducing agents with translational potential

Classical ferroptosis inducers, such as erastin and RSL3, demonstrate potent antitumor activity in preclinical models; however, their clinical translation is currently limited by unfavorable pharmacokinetic properties, including poor stability, extensive protein binding, and off-target toxicity. Nevertheless, several approved or late-stage clinical agents have been reported to induce ferroptosis indirectly and have therefore attracted considerable interest for MM therapy. Sorafenib, a multi-kinase inhibitor approved for hepatocellular carcinoma and renal cell carcinoma, inhibits system Xc^-^ at clinically achievable concentrations (72). In preclinical MM models, sorafenib depletes intracellular glutathione, enhances lipid peroxidation, and exhibits additive antitumor activity when combined with bortezomib. Sulfasalazine, a well-established anti-inflammatory agent used for rheumatoid arthritis and inflammatory bowel disease (IBD), also inhibits SLC7A11. Several small-scale studies have evaluated sulfasalazine in hematologic malignancies; however, its efficacy as monotherapy has been modest, primarily owing to limited oral bioavailability and the requirement for relatively high doses. More recently, statins, including fluvastatin and simvastatin, have been shown to reduce coenzyme Q10 (CoQ10) levels and impair the FSP1-mediated antioxidant defense pathway, thereby enhancing the activity of GPX4 inhibitors (73). Vitamin C (ascorbate) has also been investigated as a potential drug-repurposing candidate because pharmacological doses act as pro-oxidants by generating hydrogen peroxide and promoting iron-dependent lipid peroxidation (74).However, the interaction between vitamin C and bortezomib remains controversial. Preclinical studies suggest that ascorbic acid may antagonize the antitumor activity of bortezomib *in vivo*, potentially through direct interactions with boronic-acid-containing proteasome inhibitors. Consequently, current evidence does not support the use of vitamin C as a strategy for enhancing bortezomib efficacy in MM (75). Nanoparticle-based delivery systems have also been developed. Encapsulation of RSL3 or erastin within liposomes or polymeric nanoparticles improves bioavailability and reduces renal toxicity in murine models (69). Early studies have also explored GPX4-targeting proteolysis-targeting chimeras (PROTACs), which promote GPX4 degradation rather than merely inhibiting its enzymatic activity (76). Although none of these approaches has entered clinical trials for MM, several are currently undergoing early-phase evaluation in solid tumors and other hematologic malignancies. In the near term, repurposing agents such as sulfasalazine or statins in combination with established anti-myeloma therapies, together with continued optimization of drug-delivery systems, may represent a feasible translational strategy for MM.

### 6.2 Combination strategies for ferroptosis-based therapy

**Figure 3:**
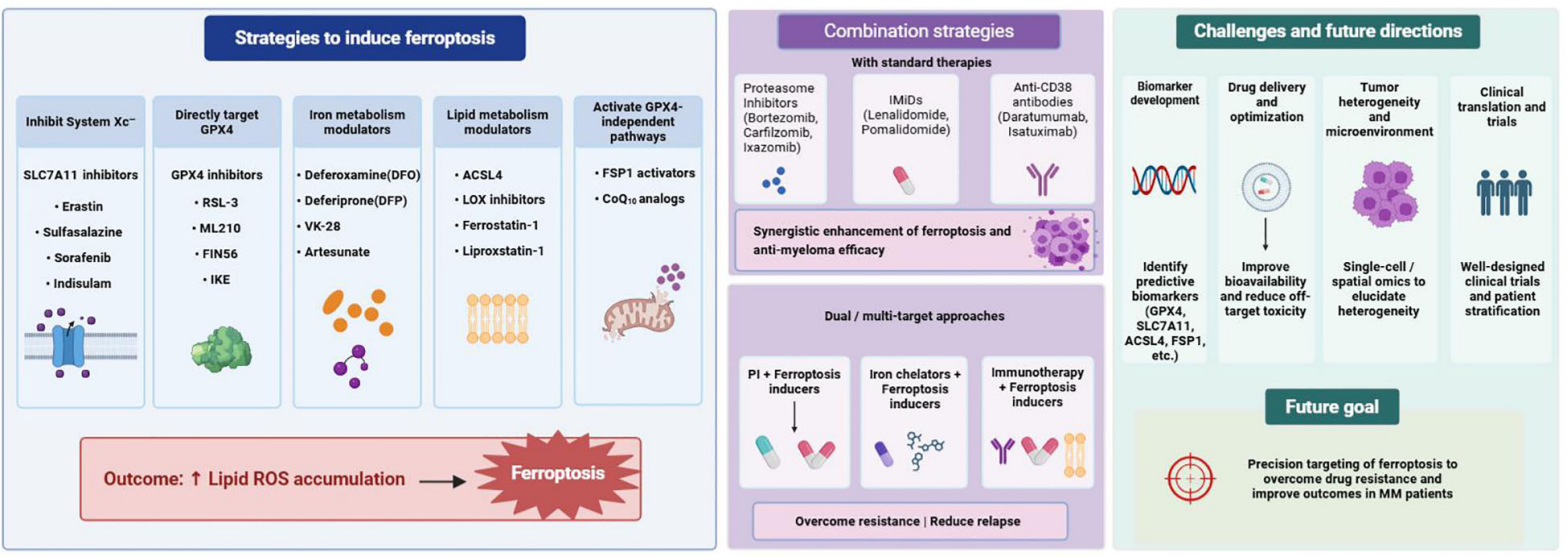
Ferroptosis-related therapeutic strategies, combination approaches, and future directions in multiple myeloma. This schematic summarizes potential approaches to modulate ferroptosis in MM, including targeting system Xc^-^/SLC7A11, GPX4-dependent antioxidant defense, iron metabolism, lipid peroxidation, and GPX4-independent protective pathways. These interventions may alter intracellular redox homeostasis and lipid ROS accumulation, thereby influencing ferroptosis susceptibility. Combination strategies with standard anti-myeloma therapies, particularly proteasome inhibitors, have shown preclinical potential to enhance anti-myeloma activity. In contrast, combinations involving IMiDs, anti-CD38 antibodies, or cellular immunotherapies remain exploratory and require MM-specific mechanistic and therapeutic validation. Future studies should prioritize biomarker-guided patient stratification, tumor-selective drug delivery, assessment of off-target toxicity, characterization of tumor and microenvironmental heterogeneity, and well-designed clinical trials. The therapeutic nodes shown are intended as a conceptual framework; most ferroptosis-targeting strategies have not yet been clinically established in MM.

Because ferroptosis inducers used as monotherapy may exhibit limited efficacy or dose-limiting toxicity, combination therapy has emerged as a more promising therapeutic strategy. MM cells exist under constitutively elevated oxidative stress, which is further enhanced by proteasome inhibitors (PIs). Consequently, the addition of ferroptosis inducers may overwhelm their antioxidant defense capacity and induce ferroptotic cell death. Preclinical evidence supports the combination of bortezomib and RSL3 in MM, whereby bortezomib enhances NCOA4-mediated ferritinophagy, increases intracellular free Fe²^+^ levels, and potentiates RSL3-induced ferroptotic stress (38). Mechanistically, PIs increase ROS production while upregulating transferrin receptor 1 (TFR1), thereby promoting intracellular iron accumulation; together, these changes predispose MM cells to ferroptosis. Combining a PI with a system Xc^-^ inhibitor depletes intracellular glutathione (GSH), inactivates GPX4, and consequently promotes extensive lipid peroxidation. In resistant MM models, this multi-target strategy effectively eliminates resistant subclones that would otherwise evade ferroptosis through FSP1 upregulation. Ferroptotic cells release damage-associated molecular patterns (DAMPs), which may activate dendritic cells and promote T-cell infiltration into the tumor microenvironment (58). Despite encouraging preclinical findings, clinical translation remains at an early stage. A phase I clinical study in patients with advanced solid tumors evaluated the combination of sulfasalazine and sorafenib, demonstrating an acceptable safety profile. In MM-related research, one ongoing clinical trial (NCT04514484), although not MM-specific, is evaluating high-dose vitamin C in combination with bortezomib. Although no dedicated ferroptosis-targeted combination trial has yet been reported in MM, several therapeutic strategies are currently under clinical consideration. Key challenges include identifying predictive biomarkers, such as baseline GPX4 expression, ACSL4 expression, and iron status, to facilitate patient stratification, as well as optimizing dosing schedules to minimize systemic toxicity (77). Given the functional redundancy of ferroptosis-defense pathways, simultaneous targeting of multiple regulatory nodes will likely be required to achieve durable therapeutic responses (78). Collectively, these findings suggest that rational combination strategies may be essential for maximizing the therapeutic efficacy of ferroptosis-based interventions in MM (**Figure 3**; **Table 3**).

**Table 3:** MM-specific ferroptosis-targeted strategies: mechanisms, combination rationale, and translational status.

Agent/strategy	Mechanism in MM	Main MM finding	Combination rationale	Evidence level	Key references
Erastin	Inhibits system Xc^-^ and limits cystine-dependent GSH synthesis.	Induced ferroptotic stress in selected MM experimental models.	May be informative for testing SLC7A11-dependent states.	*In vitro* preclinical	(54)
RSL3	Direct GPX4 inhibition.	Induced lipid-peroxide accumulation and ferroptotic death in MM models.	Potentiated by bortezomib-mediated NCOA4 ferritinophagy.	*In vitro* preclinical	(38)
Bortezomib + RSL3	Bortezomib increases NCOA4-mediated ferritinophagy and intracellular free Fe²^+^; RSL3 blocks GPX4.	Demonstrated synergistic anti-myeloma activity in preclinical models.	A mechanistically supported PI–ferroptosis-inducer combination.	MM cell-based preclinical	(38)
Class II ferroptosis inducers in t (4;14)-positive MM	Target ferroptosis-associated vulnerability in t (4;14) MM.	Showed activity in t(4;14)-positive MM models.	Supports genotype-informed ferroptosis targeting.	Preclinical	(66)
STK17B inhibition	Increases labile iron pool and lipid peroxidation.	Enhanced ferroptosis and reduced drug resistance in MM models.	May be combined with conventional anti-myeloma agents; optimal partner remains to be defined.	Cell-based and xenograft preclinical	(10)
Zalcitabine	TFAM reduction → mtDNA release → cGAS–STING activation → SLC7A11 suppression.	Induced ferroptosis in MM experimental models.	Mitochondrial stress–ferroptosis combination concept.	Preclinical	(73)
IRX4204 + lenalidomide	HMOX1–GPX4-associated modulation of ferroptotic sensitivity.	Improved lenalidomide activity in MM xenograft models.	Provides preliminary support for an IMiD–ferroptosis interaction.	Cell-based and xenograft preclinical	(85)
Andrographolide	p38/NRF2/HO-1-associated ferroptosis induction.	Induced ferroptosis in MM cell models.	Pharmacological proof-of-concept; not a clinically established MM ferroptosis therapy.	*In vitro* preclinical	(73)
Eclipta prostrata extract	Keap1/NRF2/HO-1-associated ferroptosis induction.	Induced ferroptosis in MM cell models.	Pharmacological proof-of-concept; active constituents and *in vivo* safety require clarification.	*In vitro* preclinical	(87)

### 6.3 Ferroptosis and therapeutic sensitivity/resistance in MM

Ferroptosis has emerged as a potential mechanism linking metabolic vulnerability to therapeutic sensitivity and drug resistance in MM. Proteasome inhibitors, particularly bortezomib, increase oxidative stress and disrupt protein homeostasis in MM cells. Current evidence indicates that bortezomib enhances intracellular free iron availability through NCOA4-mediated ferritinophagy, thereby increasing susceptibility to lipid peroxidation and ferroptosis (38). Accordingly, pharmacological induction of ferroptosis represents a rational strategy for enhancing proteasome inhibitor efficacy and overcoming proteasome inhibitor resistance. Available data have also implicated STK17B as a potential regulator of ferroptosis-associated drug resistance, suggesting that kinase-targeted modulation of ferroptotic signaling may provide an additional therapeutic opportunity (10). The relationship between ferroptosis and immunomodulatory drugs remains incompletely understood. Immunomodulatory drugs such as lenalidomide and pomalidomide exert anti-myeloma effects through cereblon-dependent degradation of IKZF1 and IKZF3, direct tumor-cell inhibition, and immune activation (79). Although these agents influence oxidative stress and cellular metabolism, direct evidence supporting ferroptosis as a major determinant of immunomodulatory drug response in MM remains limited. Similarly, the potential interaction between ferroptosis and anti-CD38 monoclonal antibodies remains largely unexplored. Because CD38 plays important roles in NAD^+^ metabolism, mitochondrial homeostasis, and immune regulation, combining ferroptosis-targeted strategies with anti-CD38 therapy warrants further investigation and should currently be regarded as a hypothesis requiring experimental validation (80, 81). Ferroptosis may also influence the efficacy of emerging cellular immunotherapies, although the underlying mechanisms remain incompletely understood. Lipid peroxidation and iron-dependent oxidative stress can affect T-cell persistence and cytotoxic function, whereas ferroptotic tumor cells may release immunomodulatory signals that alter antigen presentation and immune-cell activation (82). These mechanisms may be particularly relevant to CAR-T-cell therapy; however, MM-specific evidence remains preliminary (83). Overall, ferroptosis-targeted combination strategies should currently be regarded as hypothesis-driven preclinical approaches rather than established therapeutic options for overcoming drug resistance in MM. Key ferroptosis-associated mechanisms implicated in therapeutic sensitivity and resistance in MM are summarized in **Table 4**. The therapeutic value of these strategies will depend on defining the relevant resistance context, optimizing treatment sequencing and dosing, achieving tumor-selective ferroptotic stress, and preserving immune effector-cell function.

**Table 4:** Current clinical evidence and translational gaps of ferroptosis-targeted strategies in multiple myeloma.

Evidence category	Representative examples in MM	Current status	Relevance to ferroptosis	Key interpretation
Mechanistic studies	SLC7A11/GPX4-associated antioxidant regulation, NCOA4-mediated ferritinophagy, STK17B inhibition, TFAM–cGAS–STING–SLC7A11 signaling, and HMOX1/NRF2-associated redox regulation	Cell-line studies, with limited validation in primary samples or animal models	Directly demonstrate ferroptosis-associated mechanisms in MM experimental models	Provide an important biological rationale but are insufficient to establish therapeutic efficacy or safety in patients
Preclinical therapeutic studies	Bortezomib plus RSL3, STK17B-targeting approaches, zalcitabine-based ferroptosis induction, and RXR agonist-mediated HMOX1–GPX4 modulation	Cell-line and/or xenograft evidence	Support potential anti-myeloma activity through ferroptosis-related mechanisms	Require validation in clinically representative models, including bone marrow microenvironment-relevant systems, and formal toxicity assessment
Established anti-myeloma therapies	Proteasome inhibitors, immunomodulatory drugs, anti-CD38 monoclonal antibodies, BCMA-targeted therapies, and CAR-T-cell therapy	Approved or clinically investigated in MM	May interact with oxidative stress, metabolism, immune function, or ferroptosis-related pathways	These therapies should not be described as ferroptosis-targeted treatments unless ferroptosis is directly demonstrated as a clinically relevant mechanism
Ferroptosis-inducing compounds	Erastin, RSL3, iron-mobilizing or iron-loading experimental approaches, and selected natural compounds	Primarily experimental tools or preclinical candidates	Direct induction or sensitization of ferroptotic stress in laboratory models	Most compounds have unfavorable pharmacokinetic properties, limited tumor selectivity, or uncertain systemic safety
Clinical trials specifically designed to target ferroptosis in MM	No MM-specific ferroptosis-targeted interventional trial identified	No established clinical evidence	Direct clinical translation remains absent	The absence of dedicated trials represents a major evidence gap rather than evidence of clinical ineffectiveness
Biomarker-guided ferroptosis stratification	GPX4, SLC7A11, ACSL4, NRF2-related signatures, lipid-peroxidation markers, and iron-related markers	Exploratory	Potentially useful for identifying ferroptosis-sensitive subgroups or monitoring pharmacodynamic responses	No biomarker panel, analytical standard, or decision threshold has been clinically validated in MM

No MM-specific ferroptosis-targeted interventional trial identified.

### 6.4 Emerging ferroptosis-related mechanisms in MM

Recent studies have expanded our understanding of ferroptosis regulation in MM beyond the canonical SLC7A11–GSH–GPX4 axis. STK17B has emerged as a clinically relevant ferroptosis suppressor in MM. STK17B expression is increased in relapsed MM and associated with poor survival. Mechanistically, STK17B regulates iron homeostasis through IREB2 and HSPB1 while indirectly maintaining STAT3 activation, thereby limiting labile iron accumulation and lipid peroxidation. Pharmacological inhibition of STK17B increased ferroptotic stress, sensitized MM cells to conventional anti-myeloma therapies, and reduced tumor growth in xenograft models. Collectively, these findings identify STK17B as a promising therapeutic target for overcoming ferroptosis-associated drug resistance in MM (10).Autophagy-related processes may exert divergent effects on ferroptosis in MM. NCOA4-mediated ferritinophagy increases the intracellular labile iron pool through ferritin degradation, thereby facilitating lipid peroxidation. Moreover, zalcitabine-induced mitophagy has been reported to function as a compensatory survival mechanism in MM cells, whereas pharmacological inhibition of mitophagy further enhanced the anti-myeloma activity of zalcitabine. Accordingly, the therapeutic consequences of autophagy modulation may depend on the specific autophagic pathway and treatment context (84).Mitochondrial stress and innate immune signaling have also been directly linked to ferroptosis in MM. Zalcitabine downregulated mitochondrial transcription factor A (TFAM), promoted mitochondrial DNA release, activated cGAS–STING signaling, suppressed SLC7A11 expression, and induced ferroptotic cell death in MM models. TFAM overexpression or pharmacological inhibition of ferroptosis attenuated zalcitabine-induced cytotoxicity, whereas TFAM knockdown enhanced MM-cell sensitivity to proteasome inhibitors. These findings support the TFAM–cGAS–STING–SLC7A11 axis as a potential therapeutic target; however, further validation in larger patient cohorts and clinically relevant models remains necessary (84).Redox-regulatory pathways involving HMOX1 and NRF2/HO-1 have likewise been implicated in MM ferroptosis. IRX4204 activated PPARα/RXRα-dependent HMOX1 transcription while reducing GPX4 and SLC7A11 expression, thereby promoting iron accumulation, lipid peroxidation, and ferroptosis; additionally, it enhanced the antitumor efficacy of lenalidomide in xenograft models (85). In parallel, andrographolide and an ethanol extract of Eclipta prostrata induced ferroptosis in MM cells through p38/NRF2/HO-1 and Keap1/NRF2/HO-1 signaling, respectively (86, 87). Collectively, these findings suggest that HMOX1- and NRF2/HO-1-related signaling pathways exhibit context-dependent effects and remain at the preclinical stage; nevertheless, they may provide additional opportunities for ferroptosis sensitization in MM.

### 6.5 Translational challenges and current clinical evidence

Despite growing preclinical interest, ferroptosis-targeted therapeutic strategies have not yet been clinically validated in MM (88). Current evidence is predominantly derived from MM cell lines, ex vivo models, and animal studies, whereas clinical evidence supporting selective ferroptosis induction in patients with MM remains scarce. Classical ferroptosis inducers, including erastin and RSL3, have been widely used as experimental tools but exhibit unfavorable pharmacokinetic characteristics, limited tumor selectivity, and potential off-target toxicity, thereby restricting their clinical translation (89). Safety concerns represent a major barrier to the clinical translation of ferroptosis-based therapies. Ferroptosis induction may affect not only malignant plasma cells but also normal tissues vulnerable to oxidative stress and iron imbalance, including renal tubular cells, hepatocytes, cardiomyocytes, neurons, immune cells, and osteoblasts (11). his concern is particularly relevant in MM, as many patients present with pre-existing renal impairment, anemia, myeloma-associated bone disease, increased susceptibility to infection, or treatment-related organ dysfunction. Therefore, tumor-selective delivery systems, optimized dosing schedules, and rational combination strategies will be required to widen the therapeutic window. Another major challenge is the absence of clinically validated biomarkers for patient stratification. Although GPX4, SLC7A11, ACSL4, FSP1, NRF2-associated signatures, lipid peroxidation markers, and iron-related parameters have been proposed as potential indicators of ferroptosis sensitivity, no single biomarker has been clinically validated in MM (68). Moreover, the reliability of these markers may be affected by prior therapies, bone marrow microenvironmental alterations, and systemic inflammatory states. Future studies should integrate molecular profiling, functional ferroptosis assays, and patient-derived models to define clinically relevant ferroptosis-sensitive subgroups and facilitate biomarker-driven therapeutic strategies (90). Collectively, current evidence supports ferroptosis as a promising therapeutic vulnerability in MM; however, substantial translational challenges remain before ferroptosis-based interventions can enter clinical practice. Well-designed preclinical studies incorporating patient-derived models and microenvironment-relevant systems will be critical to support biomarker-guided early-phase clinical evaluation.

## 7 Conclusion and future perspectives

Ferroptosis has emerged as an area of growing interest in MM because it intersects with iron metabolism, redox adaptation, lipid peroxidation, and treatment-induced cellular stress. Current MM-specific evidence supports potential roles for NCOA4-mediated ferritinophagy, STK17B signaling, the TFAM–cGAS–STING–SLC7A11 axis, and HMOX1/NRF2-mediated redox regulation; however, the relative contribution of these mechanisms across different molecular subtypes and therapeutic settings remains to be determined. Furthermore, the bone marrow microenvironment, including stromal-cell interactions, hypoxia, iron metabolism, bone remodeling, and immune-cell function, may substantially influence ferroptosis sensitivity and should be incorporated into the design of future therapeutic strategies.

Most available evidence is derived from MM cell lines and animal models, whereas validation in primary patient samples and physiologically relevant bone marrow microenvironment models remains limited. Although ferroptosis modulation has shown promise in enhancing the efficacy of proteasome inhibitors and overcoming drug resistance in preclinical MM models, its interactions with immunomodulatory drugs, anti-CD38 monoclonal antibodies, bispecific antibodies, and CAR-T-cell therapy remain insufficiently characterized. To date, no ferroptosis-targeted therapeutic strategy has been established for the clinical management of MM.

Several challenges must be addressed before ferroptosis-based therapies can be translated into clinical practice. These include the limited tumor selectivity of currently available ferroptosis inducers, the potential for off-target toxicity affecting the kidney, bone, immune system, and other normal tissues, as well as the lack of validated biomarkers for patient selection. Future studies should prioritize clinically relevant bone marrow microenvironment models, longitudinal analyses of patient-derived samples, biomarker-guided patient stratification, and rational combination strategies with established anti-myeloma therapies. Biomarker-guided early-phase clinical studies should be pursued only after reproducible efficacy and an acceptable safety profile have been demonstrated in clinically relevant preclinical models to determine whether ferroptosis modulation can ultimately provide meaningful therapeutic benefits for patients with MM.
